# 

**Published:** 1892-02-13

**Authors:** 


					illW
'homas'B H'ospi
and NORWICH HOSPI TAL
Glasgow Wes'tern Infirmary*
with exit : nursing supplement.
price one penny.
rmu& UNfc rfcNiN Y. Iff?
^81, Vol. XI.?February 13th, 1892. FT* - JfcJ
|7J*tered at. theiQeneral Post Office aB a NewBpaper for <-?-* ?->
dismission in tho United Kingdom and Abroad. J
WITH EXTRA NURSING ^SUPPLEMENT.
Per Annum, 6s. 6d., post free.
Monthly Parts, 6d.; post free, 8d.
Ditto per Annum, 8s., post free-
No, 2St Vol. XI.] Saturday,
February 13th, 1892.
[One Pbnwi.

				

